# Many hosts, many solutions: How reservoirs shape *Borrelia* protein diversity

**DOI:** 10.1371/journal.ppat.1014583

**Published:** 2026-09-16

**Authors:** Sára Ciglbauerová, Martin Strnad, Ryan O. M. Rego

**Affiliations:** 1 Biology Centre, Czech Academy of Sciences, Institute of Parasitology, Ceske Budejovice, Czech Republic; 2 Faculty of Science, University of South Bohemia, České Budějovice, Czech Republic; Duke University School of Medicine, UNITED STATES OF AMERICA

Lyme borreliosis is the most common tick-borne infection across the Northern Hemisphere and is caused by a genetically and ecologically diverse group of spirochetes referred to as *Borrelia burgdorferi* sensu lato (s.l.) complex. Across regions, the *Borrelia* species most often associated with human disease differ: *B. burgdorferi* sensu stricto predominates among reported human infections in North America, whereas *B. afzelii* and *B. garinii* account for a large proportion of human infections in Europe and Asia [1].

It is important to note that humans are only incidental hosts and do not contribute to how these bacteria are maintained in nature. In natural transmission cycles, the *Borrelia burgdorferi* s.l. complex survives in a wide range of vertebrate hosts—from mammals and birds to lizards—each of which presents the spirochete with a distinct tissue environment, a distinct set of immune defenses, and a distinct opportunity for colonization and transmission [2].

It is tempting to view *Borrelia* diversity primarily through a clinical lens: multiple genospecies, extensive antigenic variation, and variable, often inconsistent correlations between genotype and clinical presentation. Because diagnostic assays and candidate vaccines typically target only a small number of surface antigens, this variation directly complicates efforts to diagnose, study, and prevent human disease. However, many of the traits that complicate a human-centered picture are better understood as adaptations to a more fundamental challenge: persisting in, and transmitting among, heterogeneous communities of reservoir hosts. Selection in enzootic cycles is not driven by what succeeds in a single host species, but by what can repeatedly establish infection and persist across the range of hosts that ticks feed on in nature. Because ticks acquire and transmit spirochetes through the skin during blood feeding, persistence in deeper tissues becomes relevant only after tick-borne transmission has occurred.

A recurring theme in the ecology of Lyme borreliosis is that not all *B. burgdorferi* s.l. genospecies are ecological generalists. Evidence compiled across field studies, experimental infections, and comparative analyses shows that particular *Borrelia* genospecies occur in association with particular reservoir host types far more often than would be expected by chance; i.e., these genospecies–host associations are statistically consistent and reproducible rather than random [2].

Some genospecies, such as *B. burgdorferi* s.s., use a comparatively broad range of hosts, whereas others show more pronounced, though not exclusive, host preferences. These can be broadly summarized as mammal-associated (for example, *B. afzelii*, *B. bavariensis*, and *B. spielmanii*), bird-associated (including *B. garinii* and *B. valaisiana*), and lizard-associated (*B. lusitaniae*) [2].

These associations matter because they provide a framework for interpreting the diversity of *Borrelia* surface proteins. If a given lineage is maintained mainly by a particular set of hosts, then the proteins that mediate infection and persistence in those hosts, i.e., those involved in tissue adhesion, immune evasion, and early establishment of infection, are expected to carry sequence signatures of adaptation to those hosts. This framework also helps avoid a common pitfall in Lyme borreliosis research, in which sequence differences between strains are treated as unexplained anomalies. Instead, much of this strain-to-strain variation can also be interpreted as biologically meaningful, non-random diversity shaped by which reservoir hosts drive transmission in a given region.

Below we highlight core principles linking *Borrelia* genomic architecture to protein diversity that supports vertebrate infection, illustrating how selection in reservoir hosts can maintain *Borrelia* diversity in nature.

## A genome like no other: Linear replicons and plasmid-rich coding capacity

The *Borrelia* genome is unusual among prokaryotes. Instead of a single circular chromosome, Lyme borreliae possess a linear chromosome together with a variable set of linear and circular plasmids, and this plasmid complement differs not only between species but also between strains of the same species [3,4].

This segmented genome has important consequences for host adaptation. Plasmids carry a substantial fraction of the genes involved in vertebrate infection, including many surface-exposed lipoproteins, adhesins, and immune-evasion factors [3]. Concentrating these host-interaction genes on plasmids creates a genomic architecture that could possibly facilitate diversification of precisely the genes most likely to experience strong, host-driven selection. Reservoir communities differ across geography, habitat, and season, and even within a single site the composition of competent hosts can vary substantially. In this context, the capacity to diversify, recombine, or reconfigure plasmid-borne, host-interaction genes offers a means of keeping pace with changing selective pressures. This genomic plasticity, used here to mean the variable plasmid content among species and strains, rather than the fragmented structure of the genome itself, therefore provides a mechanistic basis for how *Borrelia* lineages can maintain extensive heterogeneity in host-associated traits while remaining successful in enzootic cycles [3].

## Adhesins that match extracellular matrix landscapes for colonization and dissemination

Once inside a vertebrate host, successful infection requires more than survival in blood; it requires reliable attachment to host tissues and the ability to disseminate to niches that support persistence and onward transmission. Many *Borrelia* surface proteins act as adhesins, binding extracellular matrix (ECM) components and thereby influencing tissue tropism and colonization efficiency [5–7].

### DbpA/DbpB: Conserved function, variable implementation

Decorin-binding proteins A and B (DbpA and DbpB) illustrate how the same functional requirement of binding host connective tissue can be met by two proteins that differ markedly in their degree of sequence conservation. Molecular analyses of the *dbpA* and *dbpB* genes across the *B. burgdorferi* s.l. complex show that DbpA is substantially more variable in sequence than DbpB: DbpA sequence divergence can exceed 30% among genospecies, whereas DbpB is comparatively conserved [8]. Both proteins bind decorin and related glycosaminoglycan-containing structures and contribute to tissue colonization [5,8]. We focus on DbpA not because DbpB is unimportant as both contribute to efficient tissue binding, but because the high sequence variability of DbpA, set against the relative conservation of DbpB, makes DbpA a particularly clear example of a protein whose diversification may be shaped by differences among host tissues.

One ecological interpretation is that this sequence diversification reflects selection to “fit” the connective-tissue environments of different hosts. Vertebrate reservoirs differ in tissue composition, in the abundance of proteoglycans such as decorin, and in the structure and distribution of their glycosaminoglycan chains. If different reservoir hosts present spirochetes with distinct ECM compositions, differing both in the amino acid sequence of ECM proteins and in the glycosaminoglycan moieties attached to them, then different DbpA binding affinities or interaction modes could be favored in different hosts [9,10]. Importantly, this hypothesis does not require that each DbpA variant map neatly onto a single host species. Rather, it predicts that the composition of the local host community can maintain several co-existing DbpA variants, each effective in a subset of hosts.

### BBK32: Adhesion and immune interference in a single molecule

A second principle, illustrated by BBK32, is that some surface proteins perform several distinct functions simultaneously. BBK32 plays a role in adhesion and dissemination by interacting with ECM components such as fibronectin and glycosaminoglycans, mediating attachment to vascular and tissue surfaces that can promote dissemination and early colonization [11]. In parallel, BBK32 interferes with innate immunity by inhibiting the classical complement pathway: it binds components of the C1 complex with high affinity, and structural and functional studies have shown that it targets the activity of the C1r protease [12,13]. BBK32 thus illustrates how a single adhesin that promotes attachment and dissemination can also protect the spirochete from complement-mediated attack during the early stages of infection. Here, the “diversity” of BBK32 refers primarily to this functional versatility—its ability to bind multiple ligands and act on more than one host system—rather than to extensive sequence variation between genospecies.

Together, DbpA/DbpB and BBK32 underscore a key point: variation in adhesins is not simply a means of escaping antibody recognition (antigenic variation). Because these proteins are selected primarily for their ability to bind specific host ligands, sequence changes can alter binding to distinct ECM ligands and microenvironments, thereby influencing tissue tropism and the capacity to persist across different vertebrate reservoirs. In other words, selection acts not only on immune escape but also on the ability to engage ECM compositions and architectures that differ among host species—and even among tissues within a single host—favoring different adhesin variants in different niches.

## Immune evasion diversity tracks vertebrate immune diversity

If adhesins help *Borrelia* establish a foothold, immune evasion often determines whether that foothold is retained. The complement system is a first-line innate defense—a cascade of proteins that recognizes and destroys microbes—and its components and regulators differ between vertebrate species. These interspecies differences mean that complement can strongly influence whether a given *Borrelia* lineage survives in a particular host [14]. Although complement evasion is often described as a general virulence trait, in an ecological context it becomes a source of host differentiation: if a lineage depends on particular reservoir hosts, then its complement evasion repertoire is expected to be effective specifically against the complement systems of those hosts. A useful way to conceptualize this, particularly for host association, is to think of complement as a “gatekeeper” that determines which hosts are permissive for which spirochetes [15]. Consistent with this, comparative and ecological analyses argue that strain-to-strain variation in complement evasion helps to explain differences in host range and host association across Lyme borreliae [14]. Lyme borreliae encode several families of complement-interacting proteins, including the CRASP/CspA and OspE families; here we focus on BBK32 (elaborated upon in the above section) and CspZ as two of the best-characterized examples whose activities can be linked explicitly to host-specific complement regulators.

### Factor H interactions and host-linked tuning

Factor H (FH) is a key soluble regulator that normally protects host cells from complement attack; many microbes, including Lyme borreliae, recruit FH to their own surface to gain the same protection. FH is particularly relevant here because its sequence and regulatory activity vary across vertebrate species, and many Lyme borreliae encode FH-binding proteins. Among these, the CspZ protein (also known as BbCRASP-2) has been studied for its ligand-binding properties and its role in complement evasion through FH interactions [16].

The broader point is not merely that CspZ binds FH, but that differences in FH between host species create an evolutionary landscape in which distinct CspZ variants can be favored in different reservoir contexts. Consistent with this, inadequate FH binding has been associated with increased sensitivity to human complement, directly linking a specific molecular interaction to serum survival [17] and supporting the evolution of distinct CspZ variants, each tuned to FH of its preferred reservoir host, in different *Borrelia* genospecies. For example, CspZ from *B. garinii* (a bird-associated species) and from *B. lusitaniae* (the only lizard-associated *Borrelia* species) does not bind FH from mammalian hosts [18]. Reptilian sera, moreover, have been shown to kill many *Borrelia* genospecies through complement-mediated (borreliacidal) activity [19]. Together, these observations suggest that *B. lusitaniae* is specifically adapted to survive and even thrive in a complement environment that is lethal to most other *Borrelia* genospecies, illustrating how host-specific complement regulators can shape the distribution of complement evasion proteins across genospecies.

Viewed this way, diversity in immune evasion reflects not simply an arms race with a single host, but an ongoing arms race with the particular community of hosts that maintains a lineage in nature.

## Adaptive immune selection and persistence

### VlsE antigenic variation sustains long-term infection for transmission

For *Borrelia*, success in a reservoir host depends not only on establishing infection, but on maintaining that infection long enough for the bacteria to be transmitted to newly feeding larval ticks. The VlsE antigenic variation system is central to this long-term persistence in mammals: during infection, segmental gene conversion at the *vls* locus continuously generates new VlsE variants, allowing the spirochete to escape the host’s developing antibody response and to survive over time in immunocompetent hosts [20,21]. By prolonging infection, VlsE increases the probability that an infected reservoir host remains infectious to ticks, thereby helping to stabilize transmission within natural host communities.

### OspC as a signature of host-structured niches

Some surface proteins are under such strong immune pressure that extensive polymorphism is expected. Outer Surface Protein C (OspC) is a canonical example. Population genetic analyses show that OspC is highly diverse, and the distribution of OspC types is consistent with the idea that different vertebrate hosts represent different ecological niches that can maintain OspC diversity through balancing selection [22].

The ecological significance of OspC diversity is best stated cautiously. Beyond complicating strain typing and vaccine design, the pattern of OspC variation is consistent with a model in which host heterogeneity helps preserve antigenic variation through balancing selection. If host species differ in their immune repertoires, exposure histories, and competence for particular *Borrelia* lineages, then no single OspC type is expected to be universally optimal; instead, multiple OspC variants can be maintained because each is favored in some subset of hosts [22]. Based on this evidence, OspC diversity is better interpreted not as an anomaly but as an expected consequence of enzootic transmission through heterogeneous reservoir communities, while recognizing that the precise selective pressures acting on individual OspC types remain to be fully resolved.

## Beyond humoral immunity: Macrophage evasion and the expansion of “host-interaction space”

Although complement- and antibody-mediated pressures receive the most attention, cellular defenses may also shape selection on surface proteins, though this idea is at an earlier stage of investigation. A recent study (available as a preprint and not yet peer-reviewed) reported that the *B. burgdorferi* outer membrane protein P66 can act as a bacterial mimic at the mammalian CD47–SIRPα axis, binding SIRPα and reducing macrophage-mediated clearance [23]. We include this observation deliberately as it is a novel finding that, if confirmed, would identify a previously unrecognized route, cellular rather than humoral immunity, by which host variation could act on a Borrelia surface protein. Separately, P66 is known to contain, among its conserved hydrophobic regions, a surface-exposed loop whose sequence varies considerably across *Borrelia* genospecies [24]. Whether this loop variation influences how different genospecies interact with macrophages, or their escape from cellular immunity more broadly, remains an open question that will require dedicated experimental study.

This is important for two reasons. First, it expands the set of immune axes that can plausibly drive diversification in host-interacting proteins beyond complement and antigen recognition. Second, it reinforces the idea that *Borrelia* success depends on a multi-dimensional fit to host biology—attachment, dissemination, complement survival, and cellular immune evasion, any of which can differ among vertebrate reservoirs. Even if particular mechanisms prove more or less important in different host species, the broader inference holds: protein diversity provides the raw material for ecological flexibility.

## Re-centering Lyme biology on the enzootic cycle

**Fig 1:**
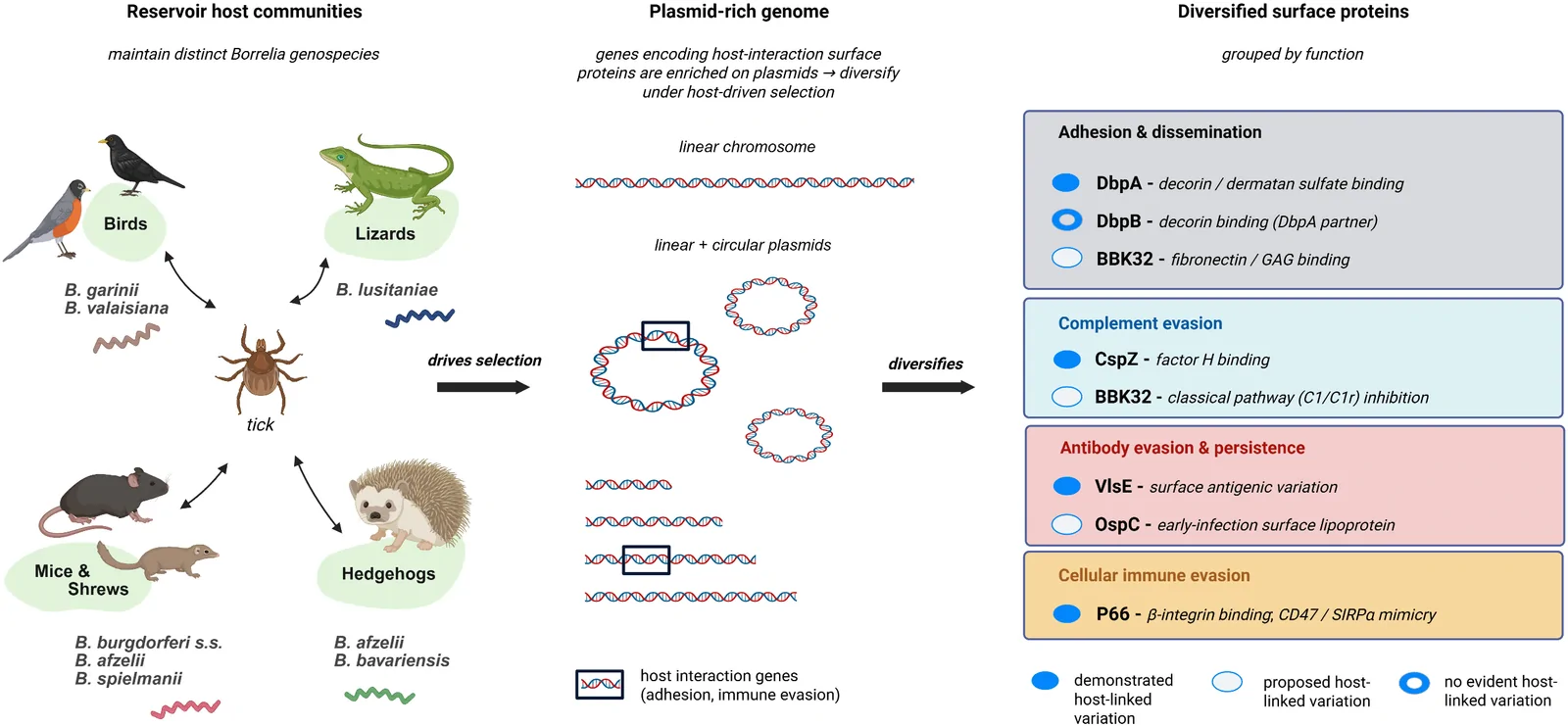
Reservoir host communities drive the diversification of plasmid-encoded surface proteins in the *Borrelia burgdorferi* sensu lato complex. (Left) *Ixodes* ticks cycle spirochetes through vertebrate reservoir communities, with distinct genospecies associating preferentially with different host groups: birds (*B*. *garinii, B*. *valaisiana),* lizards (*B*. *lusitaniae),* mice and shrews (*B*. *afzelii, B*. *spielmanii, B*. *burgdorferi* sensu stricto), and hedgehogs (*B. afzelii, B*. *bavariensis).* Double-headed arrows denote tick-mediated acquisition and transmission; several genospecies (e.g., *B*. *afzelii)* are shared across mammal groups rather than restricted to one host. (Center) Each genospecies carries a linear chromosome together with numerous linear and circular plasmids. Genes encoding host-interaction surface proteins (adhesins and immune-evasion factors, boxed area) are enriched on the plasmids, where they are subject to host-driven diversifying selection; conserved host-interaction genes such as *p66* remain chromosomal. (Right) The encoded proteins are grouped by function: adhesion and dissemination (DbpA, DbpB, BBK32), complement evasion (CspZ, BBK32), antibody evasion and persistence (VlsE, OspC), and cellular immune evasion (P66). Filled in cicles represent proven host-linked variation, empty cirlces represent proposed host-linked variation and partially filled circles represent no current evidence for host-linked variation. The P66-CD47/SIRPa interaction is currently supported only by preprint data and should be regarded as preliminary. Created in BioRender. Strnad, M. (2026) https://BioRender.com/vsl24g8.

Taken together, these observations—plasmid-rich genomes, structured host associations, diversity in ECM binding, complement “gatekeeping,” and strong selection on key antigens—support a single unifying interpretation: much of the complexity of *Borrelia* is not incidental. Rather, it reflects how enzootic transmission through diverse vertebrate hosts maintains and shapes diversity in the very proteins that mediate vertebrate infection (Fig 1). We present this as an interpretive framework and, where the evidence is still emerging, as a set of testable hypotheses rather than settled conclusions.

This has practical implications that go beyond traditional studies in ecology.

- Treat diversity as interpretable (and testable), not just descriptive. Patterns of protein variation become hypotheses about reservoir use, tissue tropism, and immune compatibility—predictions one can take back to field sampling, xenodiagnosis, and controlled infection models.
- Rethink overgeneralizing from single-host laboratory systems. A strain that is highly fit in one mammal species under controlled conditions may not represent what drives transmission in the field, where host communities vary and where ticks repeatedly encounter immune-experienced animals.
- Use protein diversity to guide translational strategy rather than just complicating it. Surface proteins remain attractive for diagnostics and interventions precisely because they are exposed and functionally important—but those same features place them under strong diversifying selection. Reviews of *Borrelia* surface proteins increasingly emphasize biomarker potential, yet ecological selection helps explain why single-marker approaches often struggle across regions, genospecies, and reservoir communities [4].

This points toward a more ecology-aligned approach to durable progress: combine multiple markers/targets, emphasize conserved functional interfaces where possible, and integrate local enzootic context (dominant genospecies, reservoir composition, and immune exposure history) into study design and interpretation. In short, the complexity of *Borrelia* is not an obstacle to be circumvented—it is the signal that reveals how these spirochetes persist in nature, why host association emerges, and why their interactions with vertebrate immunity remain so evolutionarily dynamic.
